# A Balancing Act: Partnership Dynamics in Practice When Organising and Developing Integrated Care Initiatives

**DOI:** 10.5334/ijic.9359

**Published:** 2026-02-06

**Authors:** H. C. Heek, Laura A. Nooteboom, Anne Marie Barnhoorn-Bos, Robert R. J. M. Vermeiren, Eva A. Mulder

**Affiliations:** 1 LUMC Curium – Department of Child and Adolescent Psychiatry, Leiden University Medical Center, Post Box 15, 2300 AA Leiden, The Netherlands; 2 GGZ Rivierduinen Children and Youth, Institute for Mental Health Care, Postbox 405, 2300 AK Leiden, The Netherlands; 3 Research Institute Child Development and Education, University of Amsterdam, Nieuwe Achtergracht 127, 1018 WS Amsterdam, The Netherlands; 4 Department of Child- and Adolescent Psychiatry and Psychosocial Care, Amsterdam UMC, Meibergdreef 5, 1105 AZ Amsterdam, The Netherlands

**Keywords:** integrated care, specialist care, partnership, integrated workforce, collaboration

## Abstract

**Introduction:**

Integrated care for families provides tailored, coordinated support across various life domains. It relies on partnerships between families, professionals, organisations, and policymakers, navigating diverse perspectives, cultures, and structures. These differences make partnerships complex and not a given. While much is known about partnerships, the dynamics between stakeholders in practice remains underexplored. Therefore, this study examines the dynamics of partnerships in the organisation and development of integrated care initiatives, identifying important aspects, facilitators and barriers.

**Method:**

This qualitative study explored partnership by following five integrated care teams over two years, through interviews (n = 54), observations of clinical case discussions (n = 40) and four learning sessions, incorporating perspectives of families, professionals, managers and local policymakers on partnership.

**Results:**

Four aspects of partnership were identified: shared vision among stakeholders; roles and responsibilities; monitoring and evaluation; and funding. Facilitators included inclusive participation, transparent communication, and flexible approaches. Barriers were conflicting interests, undefined roles and leadership, and fragmented systems that may hinder collaboration.

**Conclusion/Discussion:**

Balancing relational and organisational aspects of integrated care is complex yet essential to provide person-centred care. Continuous stakeholder involvement, along with evaluation and reflection, is crucial for fostering shared learning and ensuring the development and sustainability of partnerships within these initiatives.

## Introduction

Globally, there is a trend towards organising integrated care to address the mental and social needs of children and their parents (i.e. families) facing multiple and enduring problems across different life domains [1]. Integrated care can be defined as tailored and coherent support, with a broad view of families’ strengths and needs, in collaboration with involved professionals using shared decision making [1,2,3,4]. Previous studies highlight its effectiveness in countering fragmentation in care for these families [1,5,6,7]. However, organising integrated care is complicated due to the wide range of stakeholders involved, including policymakers, healthcare providers, and families, across sectors such as mental health, social care, and child protection [2,3,4,5].

These stakeholders often have different perspectives, cultures and identities, which may lead to conflicting expectations and tensions in collaboration [8,9]. These tensions reflect the dynamics of stakeholder interactions, including negotiations, alignments, and challenges that occur as families, professionals, organisations, and policymakers work together to organise integrated care. Such dynamics involve both practical coordination (e.g., defining roles, shared vision, and managing resources) and emotional dimensions (e.g., professional sensitivities and responding to families’ complex needs) [10,11]. Hence, successfully organising and sustaining integrated care requires continuous learning, reflection, and development in partnership [1,12,13,14,15].

Valentijn and colleagues [3] previously developed a framework that guides in understanding how to organise integrated care. Their ‘Rainbow model’ divides integrated care into three levels: I) practice level, centred on professionals and clients (e.g. families), ensuring continuous and collaborative care delivery [1,5,11]; II) organisation level, addressing inter alia policy development of care organisations encompassing elements like organisational cultures, leadership, and network maintenance; and III) policy level, focusing on the development of mental and social care policies, by local and national governments. An effective strategy to connect these different levels of integrated care is by establishing partnerships among involved stakeholders [3,16,17,18,22]. Partnerships can take various forms – formal or informal – and are based on the principle that no single individual or group can address complex health and social issues alone [8,19,20,21].

However, forming and maintaining partnerships when organising and developing integrated care is challenging [3,21]. It requires balancing horizontal integration (among professionals) and vertical integration (across organisations and policies), both of which present their own challenges. Horizontal integration relies on informal, interpersonal aspects like trust, shared vision and mutual benefits. Vertical integration depends on formal aspects such as financial agreements and organisational structures including monitoring and evaluation [3,22,23,24]. Moreover, there is a tension between integration and autonomy between which stakeholders must navigate in order to both collaborate effectively and maintain their independence [25]. Another difficulty is that the development of partnerships is not a linear process and its success largely depends on the context, meaning that partnerships often develop in diverse ways [3,8,9,17]. Despite the increasing focus on interprofessional partnerships and network governance in the literature [18,21,26,27,28], few studies address both the interprofessional and interorganisational aspects of partnership [29], particularly in the context of integrated care. Moreover, in practice we see that partnerships are often sought in the initial stages of setting up integrated initiatives. However, once these initiatives are operational, joint learning and further developing integrated care in partnership seems to be limited.

In the Netherlands, since 2015, youth care – from prevention to specialist care – has been decentralised to local governments. One of the main goals of this decentralisation was to achieve integrated care for families. Partnerships between local governments, care organisations, professionals, and families are essential to realise this goal. However, in practice, organising and further developing integrated care has proven to be a significant challenge. Reports highlight systemic issues such as financial constraints, fragmented interests, and inconsistent coordination between local governments and care providers. These challenges often lead to tensions within integrated care initiatives, hindering their progress and sustainability [30,31].

This qualitative study aims to explore how dynamics between multiple stakeholders within partnerships manifest in the practice of organising and developing integrated care. Specifically, we seek to identify aspects involved in organising and further developing integrated care initiatives in practice, involving multiple stakeholders, as well as the facilitators and barriers that influence these aspects. In that we mainly focus on informal, interpersonal aspects including shared vision and roles, and organisational aspects including, e.g., financing. We expect that the outcomes of this study will contribute to a deeper understanding of how partnership-based integrated care initiatives can address the challenges of organising and developing integrated care for families with multiple and enduring needs.

## Methods

### Design and setting

In this study, we followed five integrated care initiatives over the course of two years, which were active in four regions of The Netherlands: Haaglanden, Alphen aan den Rijn, Midden-Holland, and Katwijk. Each initiative included a fully integrated care team: Specialised Integrated care Teams (SITs) [16]. SITs were organised to fill a gap in the care of families with multiple and enduring needs, since multiple specialised care organisations were involved in care for these families, but often fragmented and apart from primary care services [32,33]. Therefore, this setting is particularly interesting in studying how the dynamics within partnerships manifest in the practice of organising and developing integrated care.

The SITs consisted of two to sixteen professionals from a range of specialised youth and adult care services, including mental health, parenting support, care for intellectual disabilities, and addiction care. Professionals in the SITs included social workers, psychologists, and psychiatrists who provide diagnostics, support, and treatment.

This qualitative study is part of the research project ‘De specialist dichterbij?!’ (translated: The specialist nearby?!). It reports on the findings of (I) semi-structured interviews with parents, youth, professionals, managers of organisations and local policymakers – all involved in the integrated care initiatives; (II) observations of clinical case discussions of the integrated care initiatives; and (III) learning sessions with representatives of families, professionals, care organisations and policy.

The Medical Ethics Review Board of Leiden University Medical Center has determined that this research project is not subject to the Medical Research Involving Human Subject Act (WMO) and complies with the Netherlands Code of Conduct for Research Integrity (N20.200). We applied the Consolidated Criteria for Reporting Qualitative Research (COREQ), to ensure clear and comprehensive reporting of the study methods [34].

### Participants

To better understand the dynamics within partnerships between stakeholders when organising and developing integrated care from various perspectives, our interview sample included parents (n = 18), youth (n = 3), professionals (n = 21), care organisation managers (n = 6), and local policymakers (n = 9). Recruitment for the interviews took place in collaboration with a representative from each SIT, who assisted in recruiting professionals from their respective teams. Although we tried in advance to include individuals with different levels of expertise, job functions, educational degrees, work experience and gender (purposeful sampling [35]), busy work schedules and a majority of women in the SITs made us more pragmatic. Consequently, we included all professionals who applied for an interview based on convenience sampling [35]. Parents and youth were recruited by professionals from the SITS, by handling them an information flyer that was designed in collaboration with a parent representative (CT). Four parents were interviewed in duos, they were counted as individuals. Additionally, two parents were interviewed with professional support, with the professional remaining in the background as much as possible. For this study, parents and youth were grouped under the category of ‘families’. As with professionals, we included all parents and youth who applied for an interview. The interviews with managers of care organisations involved in the SITs and local policymakers did take place using purposeful sampling; they were contacted directly by researchers EH and AB. Detailed demographic information about families, professionals, managers and policymakers can be found in Appendix A.

During weekly clinical case discussions, observed approximately every six weeks, professionals from SITs evaluated the care provided to families; the goals and needs in care of families; and the collaboration within the team and with other care organisations. The number of professionals present during these meeting usually averaged five, with the composition varying depending on the specific case being discussed.

In the four learning sessions, each SIT was represented by at least one professional, a manager of an involved organisation, and a local policymaker. Additionally, a parent representative (CT) joined each session. The sessions were led by a facilitator (CK) and attended by three researchers (EH, AB, LN), who also prepared and observed the sessions. During the final learning session, focused on partnership between families and professionals, six youth and parents with lived experiences also joined. On average, twelve participants joined the learning sessions (ranged: 8–17 participants).

The exploratory interviews, semi-structured interviews and observations were conducted over the two-year duration of the study (2020–2022), while the learning sessions took place during the final year (2022). These sessions were held quarterly, allowing participants to apply the obtained knowledge and implement the formulated actions between sessions.

### Data collection

Data collection occurred from March 2021 to April 2022. Two female researchers (EH, AB) conducted semi-structured interviews (n = 54) using a topic list based on a literature review (1) and exploratory interviews prior to this study (2020) with professionals (n = 20) from various fields (including, practice, policy, research, and education). The language use of the topic list for parents and youth was formulated in collaboration with parent representative CT. A variety of topics on integrated specialised care emerged in the interviews, as it was conducted for the broader research project. For this study, we focused on the following topics: partnerships between local government, organisations and professionals; partnerships between organisations; partnerships between professionals; partnerships between professionals and families; and the topic learning and development (see Appendix B). The interviews took place at a location (at parents/youth home, SITs location, or researchers’ office) and modality (online via MS Teams or in-person) of participants’ preference. The interviews were audio-taped, transcribed verbatim and pseudonymised by researchers (EH, AB) and students from the University of Leiden and University of Applied Sciences Leiden, under supervision of the researchers. The average length of the interviews was one hour.

Additionally, 40 semi-structured observations of the weekly clinical case discussions by the SITs were conducted by researchers (EH, AB) and three SIT practitioners, who were trained to do the observations. The observations provided us with insight into the daily activities of the SITs, offering a deeper understanding into the day-to-day practice of working in partnership. We used a pre-established observation framework, also based on the literature review and exploratory interviews, see appendix C.

Moreover, we organised four learning sessions which were part of an action learning cycle in the overall research project, organised to share preliminary results and to stimulate learning and sharing experiences among participants from different locations and initiatives. Themes that were discussed during the learning sessions included partnership between local governments, care organisations, and professionals; partnership between professionals; partnership between professionals and families; and three other themes suggested in advance by participating participants: funding of the teams; evaluation and monitoring; and the meaning of ‘specialist’ in a SIT. The learning sessions were audio-taped, transcribed verbatim and pseudonymised by the researchers (EH, AB).

Prior to the interviews and learning sessions, researchers EH and AB provided verbal information on the study (including audio recording, confidentiality, and the right to withdraw), after which participants signed informed consent forms. None of participants withdrew consent after the interview took place. Parents and youth received a gift voucher as a token of appreciation for their participation. Researchers and participants had no treatment relationship; aside from being present at clinical case observations and learning sessions, they did not know each other. Participants’ names and traceable interview information were pseudonymised. As interviews, clinical case discussions, and learning sessions were conducted in Dutch, the English translation for the quotes may affect the fluency of the sentences.

### Analysis

All interview transcripts were imported into ATLAS.ti (version 9), a qualitative data analysis software for labelling and organising text content. We applied different steps in the analysis process based on a theoretic analysis framework [37], starting with organising the data to keep track of the data and familiarise ourselves with the collected data. Second, we developed a priori topic codes tailored to the study’s purpose (deductive coding), including broad categories, as ‘collaboration between policy and practice’. It is noteworthy that the analysis for this study involved codes derived from both first and second cycle coding. Initially, for the overarching research (first cycle), codes and themes were identified, which were subsequently further analysed in a second cycle for the purposes of this study, following the steps mentioned above [36]. After that, we applied open coding to identify emerging topics and explore depth and nuance within the broader categories (inductive coding). Finally, we interpreted the data by finding patterns, recognising themes, and writing them down [36]. We analysed the observations using an observation framework focusing inter alia on partnership at all levels (see Appendix C) and the learning sessions transcripts were analysed using deductive coding.

To enhance trustworthiness, preliminary findings from interviews and observations were discussed with participants of the learning sessions. Their feedback was incorporated into the analysis, serving as a member check to refine and validate interpretations.

Moreover, we kept a reflexive attitude towards our perspectives in generating and interpreting the data by applying for strategic and contextual-discursive reflection [37]. We translated this reflexivity into practice through several approaches to ensure we remained mindful of our research objectives during data collection, analysis, and the presentation of findings (strategic reflexivity), while also recognising the influence of contextual factors and interests on individual narratives (contextual-discursive reflectivity). First, researchers EH, AB and LN applied memoing [36] while coding by maintaining a research journal for reflections on the interviews [38], and discussed coded transcripts to address any variations in coding [36]. Furthermore, regular reflective team meetings, involving researchers EH, AB, LN, and CT, further enriched this process by facilitating discussions on coding variations, refining themes, and exploring the influence of each researcher’s roles, interests, and biases from different backgrounds and expertise (AB also working clinically in youth mental health care, LN as expert on integrated care, EH conducting doctoral research on integrated care, and CT contributing from a parent experiential perspective).

## Results

### Demographics

In total, we interviewed 18 parents, 3 youth, 21 professionals, 6 care organisation managers, and 9 local policymakers. See Appendix A for an overview of the demographic characteristics of the interview participants.

### Findings

During the project, we identified four aspects of partnership that require continuous consideration to organise and further develop integrated care initiatives: (I) shared vision and transparency about interests, (II) roles and responsibilities, (III) monitoring and evaluation, and (IV) financing. Our analysis reveals that achieving these aspects in practice is not a given. The dynamics within partnerships are shaped by an interplay between stakeholders, who often bring differing interests, expectations, and levels of commitment to the table. In the following section, we first describe each aspect of partnership, followed by an exploration of how dynamics within partnership manifest in practice and the facilitators and barriers that emerge. Finally, we elaborate on the development of the four aspects over time.

#### Aspect 1: Shared vision

The first aspect we identified was achieving a shared vision and transparency about the different interests of various stakeholders involved in integrated care. In both the interviews and learning sessions, all participant groups endorsed that developing a shared vision on integrated care means a broad view, inclusion of all areas of life, continuity of care, and with that, structural engagement of all stakeholders. In that, the common thread is that the family is central in the vision.

##### Dynamics and interplay between stakeholders

A shared vision proved important to all stakeholders when organising and developing the integrated care initiative. At the case level, we see that a vision must be flexible, and an initiative needs to continuously reflect on this to remain responsive to families’ changing needs. For professionals, a shared vision contributed to a shared and supported approach, allowing them to provide this flexible and tailored care. Representatives from local governments and organisations emphasised that a shared vision fosters partnership through common ownership and responsibility. However, the dynamics surrounding the formulation and implementation of a shared vision are complex and shaped by varying levels of involvement, competition, and misaligned interests.

Interviews showed that while every stakeholder has a role in organising integrated care, the formulation of a shared vision often excludes key voices. Learning sessions highlighted that the formulation of a shared vision tends to be dominated by representatives of organisations and policy, overlooking the perspectives of professionals and families. However, professionals and families stressed that their involvement could foster mutual understanding and alignment of goals. For example, families noted that their lived experiences offer valuable insights into how care can be made person-centred. Without the input of professionals and families, a shared vision risks being abstract and disconnected from the practical challenges and complexities that professionals and families encounter daily. At the organisational and policy level, representatives emphasised that a vision developed solely at the governance level (policy and organisations) is not always effectively implemented in practice, as the ‘how’ of implementing the vision can vary depending on interests and priorities of different stakeholders. Therefore, it was emphasised that ongoing evaluation of the vision is important to adapt it to the complexities of practice. However, in practice, the focus is mostly on formulating a vision at the start of an initiative, with little attention paid to evaluating it with all stakeholders. As a result, there is an increased risk that the vision loses its focus while other interests take precedence.

“I do believe that it is important that we have a shared understanding of what is necessary. And that we also want to commit to that. So, the intentions from both sides are good and the same, and they do align. Now, we need to come together in figuring out the ‘how’.” – Local policymaker 6

##### Facilitators and barriers

Several facilitators and barriers were mentioned and observed within the development of a shared vision, displayed in Table 1.

**Table 1:** Facilitators and barriers related to shared vision.


FACILITATOR	DESCRIPTION	BARRIER	DESCRIPTION

Inclusive participation	Involving all stakeholders built trust and mutual understanding.	Competition among stakeholders	Differing expertise and priorities, especially financial, created tensions.

Transparent communication	Open dialogue aligned priorities and fostered common ground.	Exclusion of families and professionals	Top-down vision excluded key voices, causing a gap between policy and practice.

Shared commitment	Stakeholders’ willingness to collaborate enabled progress despite differences.	Misaligned interests	Diverging priorities, often financial or operational, overshadowed the vision.

Ongoing evaluation	Continuous reflection on the vision with all stakeholders.		


#### Aspect 2: Roles and responsibilities

The second aspect we identified was the delineation of roles and responsibilities among stakeholders when organising integrated care. It stresses the importance of defining each stakeholders’ role clearly from the start of an initiative and ensuring clarity on communication lines for addressing questions and challenges while providing integrated care. Moreover, it is about being familiar with other professionals and knowing others’ strengths and pitfalls.

##### Dynamics and interplay between stakeholders

Observations and learning sessions underscored the importance of clear roles and responsibilities at the practice level, particularly in interplay between professionals and families. Considering families’ experiences with multiple professionals and the diverse expertise within integrated initiatives, families emphasised that clarification of roles and responsibilities fosters trust. Without clear guidance and agreements on who provides what support, families might experience fragmented care, delays, or gaps in service. At the policy and organisational level, interviews revealed that a lack of stakeholder consensus on role division raises barriers to integration across domains in practice.

“[…] So, I believe that for these kinds of things (editor: the setup of an integrated care initiative), you need to make clearer agreements with each other. If you’re going to do a pilot, you need to define what is it going to achieve, who will take the lead, and who is responsible for what.” – Professional 19

The interviews primarily showed an interplay between stakeholders at the level of organisations and policy, potentially hindering or facilitating the delineation of roles and responsibilities. For example, different interpretations of the role of local government, as commissioner of care or as co-creator with organisations and professionals, affected the development of integrated initiatives. During the learning sessions, an interplay between policy, organisations, and practice also became visible. It showed that when integrated care initiatives were organised from practice (i.e., bottom up), but lacked organisational and policy backing, they often stall due to the absence of decision-makers for e.g., target population, goals, and methods, and responsibility for funding. Furthermore, at both the practice and case levels, unclear role divisions led to shifting responsibilities within SITs, leaving families uncertain about which professionals were responsible for specific forms of support.

##### Facilitators and barriers

The facilitators and barriers regarding roles and responsibilities are displayed in Table 2.

**Table 2:** Facilitators and barriers related to roles and responsibilities.


FACILITATOR	DESCRIPTION	BARRIER	DESCRIPTION

Regular communication	Structured contact moments streamlined communication and coordination.	Ambiguity in leadership	Lack of clear guidance left professionals uncertain about frameworks.

Local government representatives	A designated contact person promoted accountability and consistency.	Overlapping roles	Undefined responsibilities caused confusion and inefficiency in SITs.

Collaborative partnerships	Co-creation fostered shared responsibility and role clarity.	Fragmented policy support	Initiatives from practice often lacked policy/organisational backing, limiting scalability.


#### Aspect 3: Monitoring and evaluation of integrated initiatives

The third identified aspect was the monitoring and evaluation of the integrated initiatives. Monitoring involves obtaining data regarding progress and effectiveness of integrated care initiatives. Evaluation aims to learn from experiences from both the clinical case level and from monitoring data. Both processes provide insights into the care provided and how, for example, families experience it.

##### Dynamics and interplay between stakeholders

Professionals, organisational representatives and policymakers emphasised the importance of both monitoring and evaluation when organising integrated care initiatives. A common theme within the interviews, learning sessions and observations was the need for clarity on what is being monitored and why.

The development of monitoring and evaluation processes are often initiated from local governments, as they finance integrated care initiatives. Local governments often rely on robust monitoring through key performance indicators (KPIs), requiring organisations, professionals and families to provide information that illustrates the effectiveness of the integrated care provided. However, it was described challenging to quantify the outcomes of integrated care in well-defined goals and KPI’s. Additionally, during learning sessions, representatives of professionals and organisations noted that local governments sometimes posed additional monitoring questions, which evoked feelings of control and mistrust. Furthermore, different registration systems among organisations also added to the administrative burden on professionals. It was emphasised in the learning sessions that monitoring and evaluation should balance quantitative data with narratives about the experiences of families and professionals. Families, in particular, should play an active role in shaping these processes, ensuring that outcomes reflect their needs and lived experiences. When families are involved in shaping monitoring and evaluation processes, their perspectives highlight what matters in care delivery, this might lead to more relevant and personalised outcomes.

“I always ask the teams to explain how they work, and a good way to start is by looking at the numbers: how many families are we helping, and how much effort is being put in? It really makes a difference. But, most importantly, we need to keep talking in order to keep providing the care together.” – Local policymaker 3

##### Facilitators and barriers

The facilitators and barriers that were mentioned within the monitoring and evaluation of integrated care initiatives are visible in Table 3.

**Table 3:** Facilitators and barriers related to monitoring and evaluation.


FACILITATOR	DESCRIPTION	BARRIER	DESCRIPTION

Collaborative design	Early stakeholder involvement in designing monitoring and evaluation frameworks strengthened partnership and alignment.	Ambiguity in metrics	Unclear measurement goals led to misaligned priorities and tensions.

Balanced metrics	Combining KPIs with qualitative narratives gave a comprehensive view of impact.	Administrative burden	Divergent systems and reporting demands increased workload for professionals.

Open dialogue	Regular communication between stakeholders fostered trust and shared ownership of outcomes.	Feelings of control	Monitoring seen as intrusive evoked mistrust and weakened partnerships.


#### Aspect 4: Financing of integrated care initiatives

The fourth and final aspect we identified was the financing of integrated care initiatives. This entails the agreements that have been made regarding financing the initiative and how these agreements and the financial frameworks may affect the integrated care provided.

##### Dynamics and interplay between stakeholders

Financing of integrated care initiatives, frequently discussed in interviews and learning sessions, is an essential aspect within organising integrated care yet complex. Financial frameworks influenced the partnership between stakeholders and the care delivered. Two primary frameworks emerged in this study: budget allocation, where funds are distributed based on the expertise of individual organisations, and lump-sum funding, where a single sum is shared among multiple organisations. Each framework carries its own implications for partnership and care delivery.

The primary interplay in financing occurred between local governments and organisations, as organisations depend on government funding to deliver care. This dependency places organisations under pressure to demonstrate the short-term effectiveness of their interventions, which can conflict with the long-term nature of integrated care outcomes. The pressure to demonstrate effectiveness is also reinforced by market-driven funding mechanisms, causing a sense of existential uncertainty for organisations. Moreover, the different organisations involved in integrated care initiatives are often funded differently depending on their expertise. This may directly affect the care provided to families.

“It is clear there are some pretty big differences in how we approach things like recording, billing, and organising. Some organisations are laid-back, thinking, ‘Let’s just dive in and sort it out as we go.’ Others are meticulous and billing by the quarter-hour. This creates organisational gaps. So, we end up with these hiccups and tensions that just need some real talk to smooth things out.” – Representative organisation 6

From a policy perspective, funding integrated care initiatives like SITs present challenges. Policymakers acknowledged that existing fragmented care systems are often ill-suited to support the holistic and adaptive nature of integrated care. Furthermore, financial shortfalls and fixed financed periods (that were often described as too short by organisations and professionals) may hinder the ability to adequately respond to developments in practice or specific needs of families. Professionals described the added pressure this brings, as financial strains often seep into the workplace and may affect their sense of stability and focus. Families are indirectly affected by funding, primarily through the availability and accessibility of care as financial constraints can lead to gaps in services, leading to discontinuity in care for families.

##### Facilitators and barriers

Facilitators and barriers of the fourth aspect are displayed in Table 4.

**Table 4:** Facilitators and barriers related to financing of integrated care initiatives.


FACILITATOR	DESCRIPTION	BARRIER	DESCRIPTION

Flexible funding models	Adaptive financing supported the evolving needs of integrated care initiatives.	Fragmented frameworks	Differences in funding structures caused gaps in coordination and delivery of care.

Transparency in agreements	Clear and open discussions about financial expectations built trust and reduced tensions.	Short-term focus	Demand for quick results undermined the long-term nature of integrated care outcomes.

Alignment of timelines	Longer funding cycles aligned with long-term goals, providing stability for organisations and professionals, while allowing timely adjustments.	Rigid funding periods	Fixed and short financial cycles limited responsiveness to families and practice needs.


#### Development over time

Because we followed the integrated care initiatives over a period of two years, we observed that developments over time not only influenced progress within each of the four aspects but also shaped their interrelations. For example, the initiatives often started as pilots, but as they evolved, securing structural funding became essential for their continuation. This, in turn, impacted roles and responsibilities: who would take charge and make the decisions? Additionally, financial changes often led to stricter focus on monitoring outcomes, making it necessary to document effectiveness more rigorously. This often came at the expense of narrative monitoring. Furthermore, as financial and administrative priorities took centre stage, the initially shared vision sometimes faded into the background, allowing other interests to take precedence. This often affected the sense of partnership and led to tensions in the partnership between stakeholders.

## Discussion

This study explored how dynamics within partnerships manifest in the practice of organising and developing integrated care initiatives. We identified four aspects of partnership: (I) shared vision; (II) roles and responsibilities; (III) monitoring and evaluation; and (IV) financing. Although these aspects align with findings from previous studies, we found that developing these aspects in practice is not straightforward, as the interplay between the stakeholders involved can put pressure on the partnership. Effective partnerships require a balance between strategic decisions and leadership, and connection and trust among stakeholders [15]. However, previous studies often focus on initial stages of organising integrated care and organisational aspects, overlooking the importance of building and maintaining relational aspects in further developing integrated care [1,39]. Our research provides insight into how stakeholders try to find the balance between the relational and organisational aspects of integrated care.

However, this balance is often challenged by conflicting needs between the “soft” relational side of integrated care and the “hard” organisational requirements. For instance, the demand for clarity, such as clear frameworks for monitoring and evaluation, often clashes with the relational aspects of organising and developing integrated care. Our findings showed that an overemphasis on one side can hinder the development of an integrated initiative: too much focus on relational aspects may cause initiatives to stall due to for instance a lack of structural funding, while excessive focus on organisational aspects may result in partners losing connection, acting out of distrust, and ultimately cause the initiative to discontinue.

It is worth considering whether the specific context of youth care, with its fragmented structures, cross-sectoral challenges, and complex funding streams, leads to a more complicated balancing act of partnership than in other integrated care settings, such as hospitals or nationally coordinated integrated care systems.

Similarly, our findings highlight another tension that arises when there is too much focus on organisational aspects: the challenge of maintaining flexibility to meet families’ changing needs. Especially for families facing multiple and enduring needs, a flexible approach is crucial across all four identified aspects [40]. Yet, such flexibility is difficult to achieve when policies and organisations demand clear KPIs, defined frameworks and demonstrated effectiveness. Just as an overemphasis on organisational aspects can weaken relationships between partners, it can also limit the adaptability needed to provide flexible and person-centred care. Hence, integrated care requires ensuring sufficient structure and accountability while maintaining the relational and flexible approaches that are needed to align care with families.

Another important aspect of developing partnership in integrated care is critically examining the values that underpin an integrated care initiative [41], both those held by individual stakeholders and those shaping the initiative as a whole. Our study found that relational aspects are often approached in an organisational manner. For instance, a shared vision risks becoming a checklist rather than a meaningful discussion about core values such as person-centred and holistic care. When values like “effectiveness” and “goal-orientedness” take precedence, tensions between different values may arise. Future research could build on the value framework developed by Zonneveld et al [41,42,43] to explore whether these values also play a role in integrated youth care initiatives and whether the same coping strategies to tensions between values apply in this context.

Furthermore, our findings highlight the importance of involving all stakeholders from the start when organising integrated care initiatives and keeping them engaged throughout its development. However, while families and professionals play a crucial role in integrated care, their involvement is often overlooked, and the focus is mainly on policy and organisational stakeholders. Moreover, the role of local governments in integrated care is frequently unclear. They are often seen as principals, with organisations acting as contractors, a dynamic that risks widening the gap between policy and practice. Our findings suggest that integrated care not only relies on collaboration at the professional level, but also needs to be actively embedded and supported at the governance level, creating a work-climate in which partnership-based working is possible. Therefore, it is needed to organise regular moments for all stakeholders – including professionals, organizational and policy representatives, and families – to come together. Strong partnerships take time and effort, requiring both formal and informal opportunities for stakeholders to discuss differences and work toward a shared understanding [44]. Such joint interactions complement exchanges that already occur within teams or organizations and are crucial for bridging perspectives across these multiple levels [45,46]. Research underscores the significant role governance plays in integrated network collaborations, with local governments serving as key facilitators in guiding this [26]. Further research could explore the necessary role for local governments to organise integrated care initiatives in partnership with practice.

Since organising and developing integrated care initiatives is a continuous process that requires ongoing alignment between stakeholders, it is essential to keep learning and reflecting on partnership with all stakeholders [1,12,13,14,15]. However, in practice, the importance of continuous learning within an integrated care initiative is often overlooked and deprioritised. This may lead to stakeholders initially coming together to establish an integrated care initiative but gradually losing connection due to a lack of shared learning and reflection. This can hinder further development or even cause the initiative to stall, as differing interests, tensions, and shifting priorities come to the fore. Future research should explore learning approaches that facilitate collaborative learning among diverse stakeholders within integrated care initiatives. This may provide deeper insights into which forms of learning can strengthen partnerships and contribute to the development of integrated care initiatives.

### Strengths and limitations

An important strength of this study lies in its qualitative and participatory design, providing a powerful methodology for exploring complex processes in practice. We explored the complex reality of organising integrated care from partnership by combining different perspectives (families, professionals and representatives of organisations and local government) and multiple data sources (semi-structured interviews, observations and learning sessions). Through this triangulation, we intend to increase the validity of the study [47]. Furthermore, the engagement of professionals as co-researchers in the SITs and our collaboration with a parent representative during the entire study increased practical relevance of the study and its results. Moreover, discussing preliminary results during learning sessions with representatives of families, professionals, organisations and local governments enabled us to study interplay between perspectives and it served as a member check.

However, some limitations should be considered. First, the recruitment of families was done by professionals, which may have led to only selecting families they considered suitable, potentially limiting the representativeness of the target group and overlooking minority voices. To counteract this issue, we distributed a flyer to all parents and youth, encouraging direct participation. Second, qualitative research carries the risk of confirmation bias, where findings may unconsciously align with pre-existing beliefs. We minimised this through reflexive meetings during the coding and analysis process. Third, our study is focused on a specific form of organising integrated care in a specific setting. Future research could explore different forms of integrated care and examine the role of partnership within them. Fourth, our observations focused on team functioning and emerging themes of partnership, but not on interactional dynamics between stakeholders. Future research could address these dynamics more explicitly. Lastly, while our qualitative research identified four key aspects, we cannot determine their interdependence or order. Future studies, particularly longitudinal research, could track changes in these aspects over time, providing insights into their sequence and development.

## Conclusion

When organising and developing integrated care in partnerships, it is important to consider both organisational and relational aspects. Our results underscore that balancing these aspects is inherently complex. A strong focus on one side can hinder the initiative’s development, highlighting the need for continuous reflection and adjustment, with consideration for values of individual stakeholders and the integrated initiative. Furthermore, all stakeholders must be actively involved from the start and remain engaged throughout the process to ensure sustainable partnerships.

## Data Accessibility Statement

The datasets used during the current study are available from the corresponding author on reasonable request.

## AI statement

Artificial intelligence (AI) was used to support the writing process of this article. We used OpenAI’s ChatGPT (version 4) to assist with language editing, translation, and improving clarity and structure. The authors critically reviewed, edited, and approved all AI-assisted content. No AI tools were used for data collection, analysis, or interpretation. Responsibility for the content remains entirely with the authors.

## Additional Files

The additional files for this article can be found as follows:

10.5334/ijic.9359.s1 Appendix A. Demographic characteristics of the parents, youth, professionals, managers and local policymakers.

10.5334/ijic.9359.s2 Appendix B. Table B.1 and Table B.2.

10.5334/ijic.9359.s3 Appendix B. Observation framework for multidisciplinary case meetings.
