# Supplementary material for: A Balancing Act: Partnership Dynamics in Practice When Organising and Developing Integrated Care Initiatives
Source: Int J Integr Care. 2026 Feb 6;26(1):5. doi: 10.5334/ijic.9359 (PMC12880003; doi:10.5334/ijic.9359)
Supplement: Appendix B. — Table B.1 and Table B.2. [file ijic-26-1-9359-s2.pdf]

Appendix B

Table B.1
Topic list semi-structured interview youth and parents

Topic	Question
Family characteristics	Gender
	Age
	Family status and composition
	Educational level
	Type of problems (global description)
	Number of years of provided care to the family
	How did you arrive to the SIT?
	What care trajectory preceded this?
Aims and goals	What care do you receive from the SIT?
	Does the provided care meet the needs of your family?
	What are the most important goals of care for you?
	What do you find important in the support you receive?
	How do you and the professional ensure that the goals are achievable and match what is important to you and your family?
Experiences with the SIT	What is your experience so far with the SIT?
	How do you view the provided care?
	What makes this care different from the support you received before?
	What do you think could improve the care provided by the team?
Broad view of family functioning	What life domains and which family members does the SITs care focus on?
	Do you feel that the needs of all family members have been properly taken into account?
Collaborative relationships with the family	How do the team's professionals manage to build collaborative relationships with the individual family members as well as with the family as a whole?
Continuity of care	Do you feel that the right help is available at the right time?
	Do you feel that care can be provided flexible when needed?
Social network	How is your social network involved?
	What are your wishes or needs in this?

Table B.2

Topic list semi-structured interview professionals, managers and policy makers

Topic	Question
Demographics and expertise professionals	Gender<br>Age<br>Educational level<br>Number of years of work experience<br>Organisation and expertise
Aims and goals	What are the aims and focus of the SIT?<br>What should the SITs support to families with multiple, severe and enduring problems provide?<br>How does the SIT ensures that the goals of support are achievable and match what a family considers important?
Family characteristics	For which families is the SIT intended?<br>And for which families not?<br>How can families addressed by the SIT can be characterised?
Experiences with the SIT	What in the SITs working method works well or less well for this group of families?<br>What in the SITs working method do you consider an additional value to traditionally organised care?
Broad view of family functioning	What life domains and which family members does the SITs care focus on?<br>How does the SIT take the needs of all family members into account?
Collaborative relationships with the family	What is important in building collaborative relationships with these families?<br>How do you manage to build these relationships and do these families require different skills from you as a professional?
Shared decision-making	How do you and the family make shared decisions, for example about the goals of support?<br>What do you think are important elements in shared decision making with these families?
Continuity of care	How is the continuity of care for the family guaranteed (during and after the SITs support)?<br>What is the SITs role in care coordination?
Social network	How is the social network of the family involved by the SIT?
