# Supplementary material for: A Balancing Act: Partnership Dynamics in Practice When Organising and Developing Integrated Care Initiatives
Source: Int J Integr Care. 2026 Feb 6;26(1):5. doi: 10.5334/ijic.9359 (PMC12880003; doi:10.5334/ijic.9359)
Supplement: Appendix B. — Observation framework for multidisciplinary case meetings. [file ijic-26-1-9359-s3.pdf]

Appendix C

Observation framework for multidisciplinary case meetings

SIT:
Date:

Characteristics of the case	Reason/question/aim of case discussion<br>Characteristics of the family in case: family composition, age of children, type of problems, requests for support, etc.<br>Support from the SIT being provided to the family<br>Disciplines and expertise provided from the SIT<br>Forms of support and frequency
Aims of care	Focus of support regarding family members and life domains<br>Aims and goals of support<br>Clarity of goals of care
Problem analysis/ explanatory analysis	Identified problems<br>Interrelatedness of problems<br>Agreement of professionals and family on the explanatory analysis of problems
Decision-making and prioritization	Process of setting goals and prioritizing support<br>Family's needs in decision-making
Monitoring/evaluation of support	Monitoring and evaluation of the care process by professionals and the family
Scaling up and down care in and outside the SIT	Reasons and plans for upscaling and downscaling care<br>Process of upscaling and downscaling care
Terminating the care process	Reasons for terminating the care process.<br>Role and vision of professionals and family in terminating care
Role of professionals	Clarity and discussion about the role and commitment of each professional in a family's care process
Preconditions for providing appropriate support to the family	In time, finances, availability of support
Exchange of information	Availability of information on the family from previous care<br>Exchange of information between professionals
Collaborative relationships with the family	Involvement of family members in the care process<br>Family's control in the care process and in decision-making
Interprofessional collaboration in this case	Involvement of SITs professionals and expertise<br>Interprofessional collaboration around the family
Collaboration with the care network	Other care services involved in the family<br>Collaboration of the SIT with professionals and services in the care network outside the SIT<br>In referral to the SIT, during the SITs care process and at the termination of the SITs care or transfer to other services
Reflections or other	Other facilitators and barriers in the SITs working method with this

observations	family<br>Reflections on the case meeting
